# An Unusual Case of Left Atrial Mural Thrombus following Aortic Valve Replacement

**DOI:** 10.1155/2019/5254164

**Published:** 2019-04-09

**Authors:** Mohamed E. Taha, Ammar Eljack, Hisham Ibrahim, Chanwit Roongsritong

**Affiliations:** ^1^Department of Internal Medicine, University of Nevada, Reno, NV, USA; ^2^Department of Internal Medicine, Beaumont Health, Detroit, MI, USA; ^3^Department of Internal Medicine, University of Iowa, IA, USA; ^4^Department of Heart and Vascular Health, Renown Regional Medical Center, Reno, NV, USA

## Abstract

The left atrial thrombus is a well-known complication of atrial fibrillation and rheumatic mitral valve disease and carries a high risk for systemic thromboembolism. They are generally dissolved after a certain period of optimal anticoagulation. A large thrombus, on the other hand, may persist even with adequate anticoagulation. The surgical removal of a thrombus theoretically poses some risk of systemic embolization, making its management a clinical dilemma. Furthermore, a refractory thrombus is uncommon. Thus, an evidence-based guideline in selecting the optimal therapy is needed. We report a case of a 74-year-old male with atrial fibrillation and a history of unprovoked pulmonary embolism who was incidentally found to have a massive left atrial thrombus shortly after discontinuing warfarin about 4 months following bioprosthetic aortic valve replacement. The thrombus was refractory to anticoagulation posing a clinical management dilemma. This case is interesting in terms of presentation and the approach to diagnosis and treatment.

## 1. Introduction

Left atrial thrombus (LAT) formation is a well-known complication of atrial fibrillation and rheumatic mitral valve disease. They carry a high risk for systemic thromboembolism; therefore, early detection and treatment should be established with a high index of suspicion [1]. The left atrial appendage is the most common location of the LAT; therefore, the appendage is usually ligated during open heart surgery. In terms of treatment, anticoagulation remains the preferred approach once LAT has been detected. We present a case of a 74-year-old male who developed a massive LAT shortly after discontinuing warfarin about 4 months following bioprosthetic aortic valve replacement. The thrombus was also refractory to anticoagulation posing a clinical management dilemma.

## 2. Case Description

The patient is a 74-year-old Caucasian male with a history of atrial fibrillation, CHA2DS2-VASc score of 6, unprovoked deep venous thrombosis, and pulmonary embolism on long-term warfarin, who was initially found to have aortic stenosis (AS) in 2015 during preoperative cardiovascular evaluation for surgery on his right foot. His echocardiography at the time revealed moderate aortic stenosis (peak gradient of 32 mmHg, mean gradient of 22 mmHg), an ascending aorta diameter of 3.7 cm, and a severely enlarged left atrium (left atrial volume index of 66 mL/m^2^). His atrial fibrillation was controlled with propafenone and warfarin. Subsequently, his AS was followed clinically and echocardiographically every 6-12 months according to the guidelines.

By the end of 2017, he developed a worsening dyspnea on exertion and persistent atrial fibrillation along with episodes of symptomatic bradycardia (heart rate in 30-40 s) for which he underwent pacemaker implantation. His echocardiography revealed worsening aortic stenosis; the calculated valve area was 0.8 cm^2^ with a peak gradient of 45 mmHg and a mean gradient of 27 mmHg. The left ventricular systolic function was mildly reduced with an ejection fraction (LVEF) of 40%.

Upon further evaluation which included transesophageal echocardiography (TEE) and dobutamine stress echocardiography (DSE), it was felt that his clinical features were consistent with a low-flow, low-gradient severe AS. He was subsequently referred for evaluation for transcatheter aortic valve replacement (TAVR).

**Figure 1:**
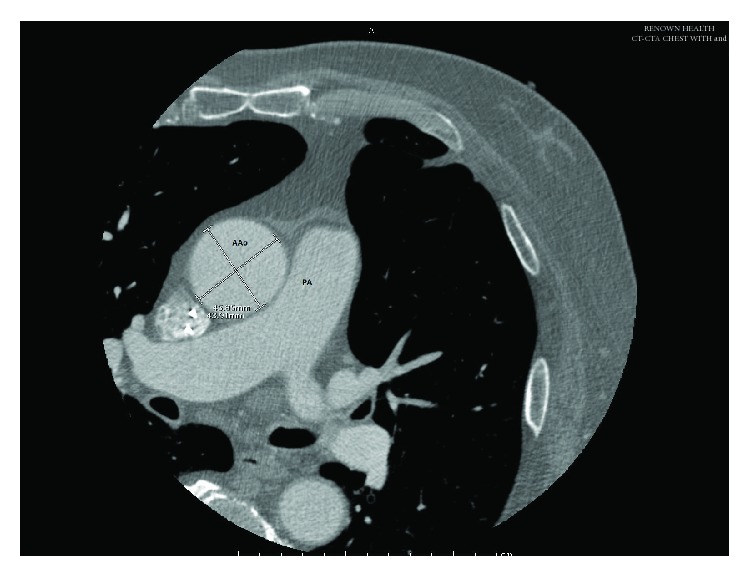
Computed tomography (CT) of the chest showing the enlarged ascending aorta measuring 4.6 × 4.3 cm. AAo, ascending aorta; PA, pulmonary artery.

While awaiting TAVR, his symptoms continued to progress as he developed syncopal episodes. Furthermore, as part of his pre-TAVR evaluation, he underwent CT angiography of his chest which revealed a worsening of his ascending aortic aneurysm with an aortic root diameter measuring 4.6 cm (Figure 1). A shared decision was made to let him undergo open heart surgery to repair both pathologies. By February 2018, he underwent a successful complex surgical procedure with bioprosthetic AVR (27 mm Edwards Perimount Magna pericardial valve), ascending aortic aneurysmal repair (30 mm Hemashield tube graft), mitral valve repair (36 mm Edwards flexible annuloplasty), left-sided maze procedure, and left atrial appendage excision and ligation (the LAA was ligated at its base and excised, and the stump was oversewn in 2 layers using #4-0 prolene sutures). He was placed back on warfarin and aspirin. He was discharged after an uneventful hospital course with referral to our outpatient anticoagulation clinic and cardiac rehabilitation program.

**Figure 2:**
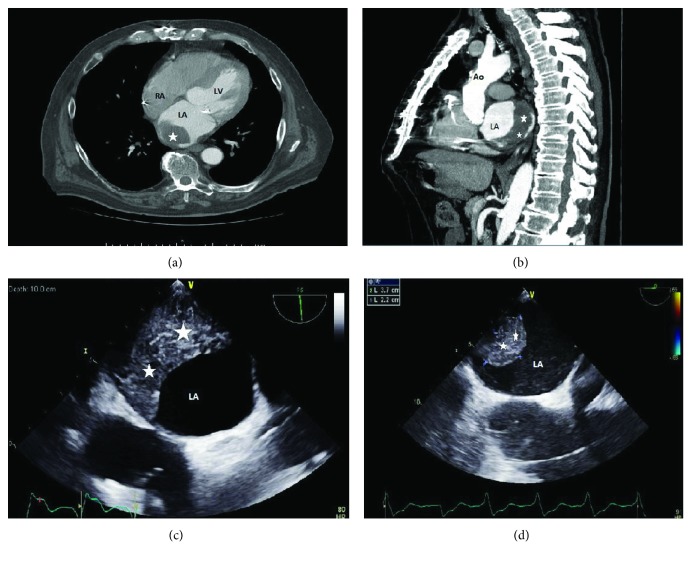
(a and b) Computed tomography (CT) of the chest showing a thrombus (stars) in the left atrium of the heart (LA). (c and d) Transesophageal echocardiogram (TEE) showing the thrombus in the left atrium of the heart (LA). Ao, aorta; LA, left atrium; LV, left ventricle; and RA: right atrium.

His anticoagulation was closely monitored. Four months later, however, he presented with persistent frank hematuria. A shared decision was made to stop his warfarin since it had been more than 3 months from his bioprosthetic valve replacement and more than 10 years from the onset of his lone PE. He was subsequently referred for further urological workup. Two months later, while his hematuria had resolved, it was accidentally discovered that he had a sizable left atrial thrombus upon undergoing surveillance CT chest imaging for his ascending aorta, which was further delineated using TEE (Figure 2). Subsequently, he was restarted back on warfarin with a heparin bridge, while no decision was made to pursue surgery.

He had a follow-up TEE 4 months later which showed a very little to no change in the size of the thrombus despite adequate anticoagulation. Fortunately, there has not been any thromboembolic event up to date.

## 3. Discussion

A refractory left atrial thrombus is a clinical dilemma because of its risk of systemic complications and a lack of an evidence-based guideline in selecting optimal therapies. LAT is often associated with atrial fibrillation or rheumatic mitral valve stenosis. They account for >45% of cardiogenic thromboembolic events [1]. LAT often forms in the left atrial appendage (LAA) because of its shape and the presence of trabeculations. However, it can arise around the free atrial wall especially in cases of a dilated atrium. In our presented case, his left atrium was severely dilated; however, the left appendage was already excised. Based on the interrogation of his pacemaker, he had also been in sinus rhythm since the day of his pacemaker implantation.

Thrombus formation in the left atrium after LAA exclusion has been previously reported with endocardial occlusion devices (Watchman, Boston Scientific, Marlborough, Massachusetts or Amplatzer Cardiac Plug (ACP), St. Jude Medical, St. Paul, Minnesota). Its mechanism was mainly attributed to platelet aggregation in the setting of a foreign body in the left atrium [2, 3]. In the study by Lakkireddy et al., the risk for LA thrombus formation using a lariat device was discovered to be as low as 2%, mostly occurring within 90 days [4]. There are no published data regarding LA thrombus formation at postsurgical excision and ligation of the LAA as in our case.

Our patient developed a massive thrombus despite taking both warfarin and aspirin. He had a history of atrial fibrillation but was in sinus rhythm for at least 6 months before the thrombus was detected (since the maze procedure). He is 74 years old, but he has no history of hypertension or diabetes mellitus. He did have one episode of DVT and pulmonary embolism along with a severely dilated left atrium which may have partly contributed to the development of the thrombus [5].

In terms of management, various options including anticoagulation, thrombolytic treatment, endovascular intervention, and open surgery exist. Anticoagulation is generally considered as the first-line therapy. Even with the failure of medical management, there is little evidence either in favor of or against aggressive management to remove the thrombus [6]. In our patient, he was restarted on warfarin and surgical consultation was made; however, the latter was deferred.

## 4. Conclusion

The left atrial thrombus is a known complication of atrial fibrillation and rheumatic mitral valve disease, especially in the setting of an enlarged left atrium. If not detected and properly treated, it can lead to devastating thromboembolic complications. Anticoagulation is usually the treatment of choice which usually results in the desolation of the thrombus; however, in some cases, LAT might be refractory to anticoagulation creating a decisional dilemma as in our presented case. More cases and effort are needed to have a standardized approach to treat this category of patients.

## References

[1] Al-Saady N. M., Obel O. A., Camm A. J. (1999). Left atrial appendage: structure, function, and role in thromboembolism. *Heart*.

[2] Gasparini M., Ceriotti C., Bragato R. (2012). Huge left atrial thrombus after left atrial appendage occlusion with a Watchman device. *European Heart Journal*.

[3] Cruz-Gonzalez I., Martín Moreiras J., García E. (2011). Thrombus formation after left atrial appendage exclusion using an Amplatzer cardiac plug device. *Catheterization and Cardiovascular Interventions*.

[4] Lakkireddy D., Vallakati A., Kanmanthareddy A. (2015). Left atrial thrombus formation after successful left atrial appendage ligation: case series from a nationwide survey. *Journal of the American College of Cardiology*.

[5] Kristiansen A., Brandt L., Agoritsas T. (2014). Applying new strategies for the national adaptation, updating, and dissemination of trustworthy guidelines: results from the Norwegian adaptation of the Antithrombotic Therapy and the Prevention of Thrombosis, 9th Ed: American College of Chest Physicians Evidence-Based Clinical Practice Guidelines. *Chest*.

[6] Egolum U. O., Stover D. G., Lenihan D. (2013). Intracardiac thrombus: diagnosis, complications and management. *The American Journal of the Medical Sciences*.

